# Bladder cancer: do we need contrast injection for MRI assessment of muscle invasion? A prospective multi-reader VI-RADS approach

**DOI:** 10.1007/s00330-020-07473-6

**Published:** 2020-11-19

**Authors:** Andrea Delli Pizzi, Domenico Mastrodicasa, Michele Marchioni, Giulia Primiceri, Francesca Di Fabio, Roberta Cianci, Barbara Seccia, Barbara Sessa, Erica Mincuzzi, Martina Romanelli, Pietro Castellan, Roberto Castellucci, Antonella Colasante, Luigi Schips, Raffaella Basilico, Massimo Caulo

**Affiliations:** 1 Department of Neuroscience, Imaging and Clinical Sciences, “G. d’Annunzio” University, Via Luigi Polacchi 11, 66100 Chieti, Italy; 2 ITAB - Institute of Advanced Biomedical Technologies, “G. d’Annunzio” University, Chieti, Italy; 3 Department of Radiology, Stanford University School of Medicine, Stanford, CA USA; 4 Department of Medical, Oral and Biotechnological Sciences, Urology Unit, SS Annunziata Hospital, G. d’Annunzio University of Chieti, Chieti, Italy; 5 Laboratory of Biostatistics, Department of Medical, Oral and Biotechnological Sciences, Chieti, Italy; 6 Surgical Pathology Unit, SS Annunziata Hospital, Chieti, Italy

**Keywords:** Bladder cancer, Magnetic resonance imaging, Diffusion magnetic resonance imaging, Contrast media

## Abstract

**Objectives:**

(1) To investigate whether a contrast-free biparametric MRI (bp-MRI) including T2-weighted images (T2W) and diffusion-weighted images (DWI) can be considered an accurate alternative to the standard multiparametric MRI (mp-MRI), consisting of T2, DWI, and dynamic contrast-enhanced (DCE) imaging for the muscle-invasiveness assessment of bladder cancer (BC), and (2) to evaluate how the diagnostic performance of differently experienced readers is affected according to the type of MRI protocol.

**Methods:**

Thirty-eight patients who underwent a clinically indicated bladder mp-MRI on a 3-T scanner were prospectively enrolled. Trans-urethral resection of bladder was the gold standard. Two sets of images, set 1 (bp-MRI) and set 2 (mp-MRI), were independently reviewed by four readers. Descriptive statistics, including sensitivity and specificity, were calculated for each reader. Receiver operating characteristic (ROC) analysis was performed, and the areas under the curve (AUCs) were calculated for the bp-MRI and the standard mp-MRI. Pairwise comparison of the ROC curves was performed.

**Results:**

The AUCs for bp- and mp-MRI were respectively 0.91–0.92 (reader 1), 0.90 (reader 2), 0.95–0.90 (reader 3), and 0.90–0.87 (reader 4). Sensitivity was 100% for both protocols and specificity ranged between 79.31 and 89.66% and between 79.31 and 83.33% for bp-MRI and mp-MRI, respectively. No significant differences were shown between the two MRI protocols (*p* > 0.05). No significant differences were shown accordingly to the reader’s experience (*p* > 0.05).

**Conclusions:**

A bp-MRI protocol consisting of T2W and DWI has comparable diagnostic accuracy to the standard mp-MRI protocol for the detection of muscle-invasive bladder cancer. The experience of the reader does not significantly affect the diagnostic performance using VI-RADS.

**Key Points:**

*• The contrast-free MRI protocol shows a comparable accuracy to the standard multiparametric MRI protocol in the bladder cancer muscle-invasiveness assessment.*

*• VI-RADS classification helps non-expert radiologists to assess the muscle-invasiveness of bladder cancer.*

*• DCE should be carefully interpreted by less experienced readers due to inflammatory changes representing a potential pitfall.*

## Introduction

Bladder cancer (BC) is a leading cause of cancer-related death in men and accounts for approximately 550,000 new cases per year worldwide [1]. About a quarter of BCs are muscle-invasive (MIBC). MIBCs are > T1 tumors requiring radical cystectomy, with or without neo- or adjuvant chemotherapy, and have significant impact on the patient survival [2]. On the other hand, non-muscle-invasive bladder cancers (NMIBCs), representing Ta–T1 tumors, are frequently treated locally and are characterized by a better prognosis [3]. Although trans-urethral resection of bladder (TURB) represents the gold standard for BC local staging, it has some limitations. In fact, it is burdened with a high risk of under-staging in the presence of multiple or large lesions and in T1 tumors potentially requiring a second TURB, causing a delay in further treatments and overall increased healthcare costs [4–6].

Multiparametric magnetic resonance imaging (mp-MRI) has recently become an emerging method for the BC local staging and, in particular, for the detection of MIBC [7–9]. In this context, the Vesical Imaging-Reporting and Data System (VI-RADS), introduced in 2018, represents a standardized reporting criterion for bladder MRI aiming to improve the communication among doctors, thus potentially improving patient management [8]. In fact, recent studies demonstrated high accuracy of VI-RADS for discriminating MIBC and NMIBC with a good inter-reader agreement [10–14]. More in detail, the recommended image protocol includes conventional T2W-weighted images (T2W), diffusion-weighed imaging (DWI), and dynamic contrast-enhanced (DCE) imaging. Among these sequences, DWI was considered the dominant sequence to estimate the muscle invasion [8]. Recent studies demonstrated the feasibility of contrast-free MR protocols for the local staging of several tumors, including rectal cancer and prostate cancer, with promising results. However, to the best of our knowledge, no studies compared the standard mp- and a contrast-free MRI protocol (for the VI-RADS assessment) [15, 16]. In this setting, scan time and costs would be reduced. Moreover, patient safety and comfort would be improved, avoiding the risks related to the intravenous administration of gadolinium-based contrast agents, such as nephrogenic systemic fibrosis, renal failure, and tissue accumulation of gadolinium.

We hypothesized that a non-contrast, biparametric magnetic resonance imaging (bp-MRI) might be useful to assess BC local staging. The purpose of this prospective study was to investigate whether a contrast-free bp-MRI, including T2W and DWI, can be considered an accurate alternative to the standard mp-MRI for the muscle-invasiveness assessment of BC, using a 3-T MR scanner. Moreover, we explored how these two different protocols affect the diagnostic performance when they are assigned to four readers with different expertise in abdominal imaging.

## Materials and methods

### Patient population and study design

**Fig. 1:**
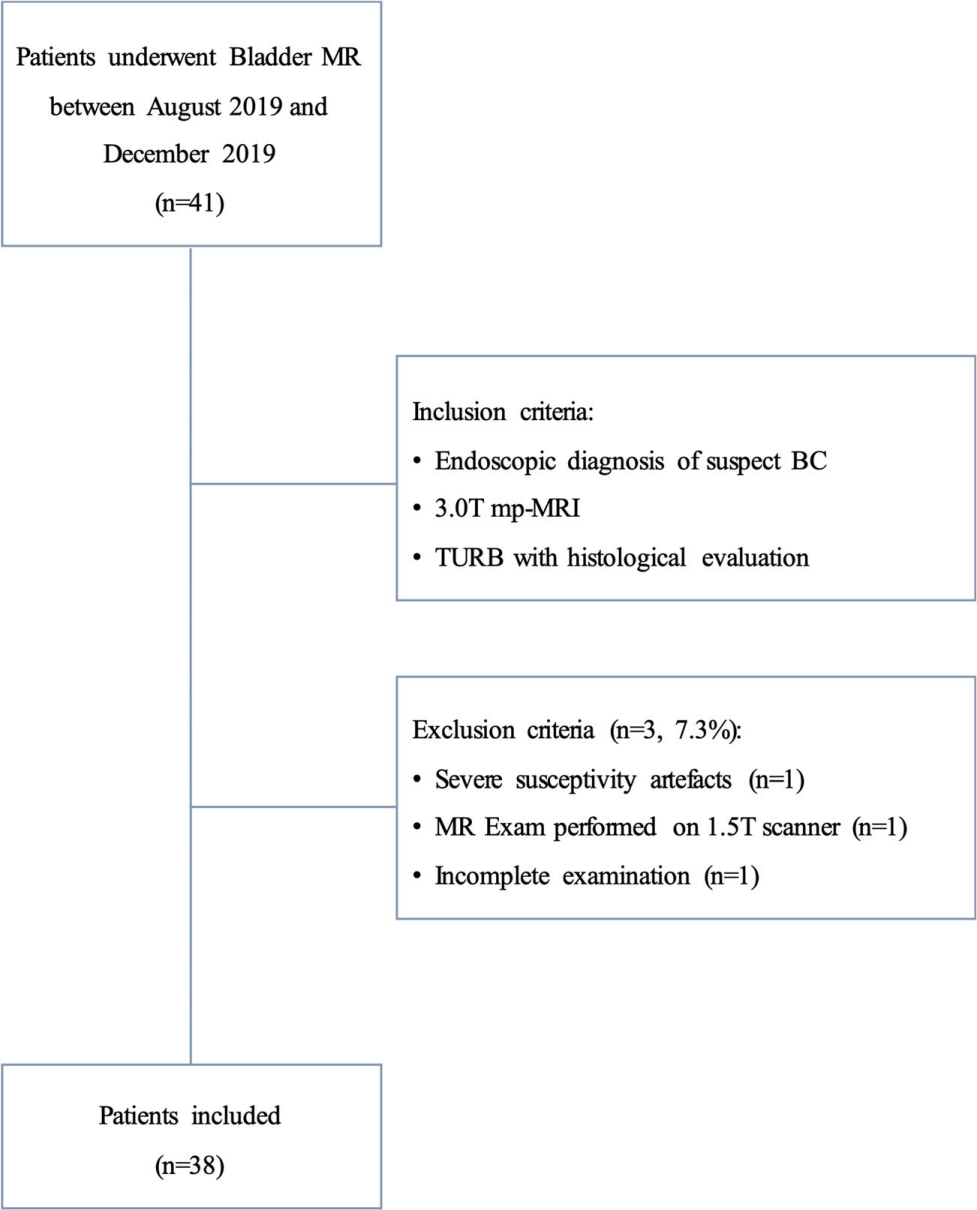
Study flowchart

This prospective study received formal Institutional Review Board and Ethical Committee approval. The study was conducted in line with the European Urology and Good Clinical Practice guidelines according to ethical principles laid down by the latest version of the Declaration of Helsinki. Written informed consent was obtained from all patients enrolled in the study [4]. A total of 41 patients who underwent clinically indicated multiparametric MRI (mp-MRI) between August 2019 and December 2019 were prospectively included (Fig. 1). Inclusion criteria were (1) biopsy-proved urothelial BC after cystoscopy, (2) mp-MRI of bladder performed using a 3-T scanner, and (3) TURB with histological evaluation. Finally, 3 patients were excluded: one patient for severe susceptibility artifacts in the pelvis (hip replacement), one because the MR exam was performed on a 1.5-T scanner, and another patient had contraindications to intravenous contrast administration. The final study population consists of 38 patients.

### MRI protocol

A state-of-the-art 3-T MR scanner (dStream, Philips Medical System) equipped with a phased array surface coil was used in this study. The MR protocol included the following sequences: T2W turbo spin-echo images, DWI, and DCE T1-weighted 3D spoiled gradient-echo images (DCE). A more detailed description of the MR parameters is summarized in Table 1. Concerning DWI, four *b*-values (*b* = 600, *b* = 1000, *b* = 1500, and *b* = 2000) were acquired and apparent diffusion coefficient maps (ADC maps) were calculated for each patient. Gadoteridol (Prohance; Bracco) was used in a dose of 0.2 mmol/kg (flow rate of 2 mL/s). Patients without contraindications received 20 mg of scopolamine butylbromide (Buscopan, Boehringer Ingelheim) intravenously to reduce the incidence of motion artifacts due to bowel motility. Patients were instructed to void 1–2 h before imaging or to start drinking 500–1000 mL of water in the 30 min before the examination, depending on the patient’s tolerance level. The degree of bladder filling was evaluated using ultrasound before the patient enters the MR room. MR was performed with patient in supine position [8].

**Table 1:** Parameters of the T2-weighted, DWI, and DCE sequences included in the study protocol

	T2-weighted fast spin-echo	DWI*	DCE
Image set 1	Yes	Yes	No
Image set 2	Yes	Yes	Yes
Repetition time (msec)	3000–5000	3000	3.1
Echo time (msec)	80	97	1.42
Matrix	200 × 179	68 × 54	100 × 139
Flip angle	90	90	10
Number of excitations	2	3–12	2
Section thickness (mm)	4	4	4
Imaging planes	Transverse^†^, coronal, sagittal	Transverse†, sagittal	Transverse^†^
Acquisition time (min)	2.26	4.19	3.26

DWI sequences included ADC map calculation

*DWI performed with *b*-values of 0, 600, 1000, 1500, and 2000 s/mm

^†^Transverse plane angulated perpendicularly to long axis of bladder

### Image analysis

Two sets of images, namely set 1 and set 2, were evaluated on a dedicated post-processing workstation (Vue Picture Archiving and Communication System, version 12.2, Carestream Health). Set 1 (non-contrast protocol) was presented in the first reading session and included the biparametric MRI (bp-MRI) protocol consisting of

- Axial, sagittal, and coronal T2W images;
- Axial and sagittal DWI images (including the corresponding ADC maps).

Set 2 (full protocol) was assigned to the second reading session and included set 1 images in addition to DCE images. Both sets of images were independently reviewed by four readers with different degrees of experience in abdominal radiology (2 expert radiologists with 10 and 15 years respectively in abdominal radiology, 1 senior resident, and 1 second-year resident), in two reading sessions separated by at least 4 weeks, to avoid recall bias. MRI criteria for muscle-invasiveness were assessed according to VI-RADS and bladder subdivisions in sectors [8, 14]. In this way, twelve sectors were considered. According to VI-RADS, all detected lesions were scored on a 5-point scale based on the likelihood of muscle invasion: 1, highly unlikely; 2, unlikely; 3, equivocal; 4, likely; 5, very likely [8]. More in detail, the presence of muscle invasion was suspected on the basis of the following criteria:

- T2W: interruption of low signal intensity line of the muscular layer (likely) or extension of the intermediate signal intensity tumor to the extra-vesical fat (very likely);
- DWI: focal extension of high signal intensity tumor on DWI and low signal intensity on ADC to the muscular layer (likely) or to the entire bladder wall and extra-vesical fat (very likely);
- DCE: focal extension of tumor early enhancement to the muscular layer (likely) or to the entire bladder wall and extra-vesical fat (very likely) [8].

### Reference standard

The urologic evaluation included the description of lesion number, size, morphology, and location. A standardized form with a descriptive schematic map was used for cystoscopy, MRI, and TURB, to record all the information on each lesion. More in detail, according to Marchioni et al, bladder was divided in ten regions: trigone, right ureteral orifice, left ureteral orifice, right wall, left wall, anterior wall, posterior wall, dome, neck, and posterior urethra [14]. All patients underwent a standard TURB and a piecemeal resection in fraction [4, 10, 11, 17–20]. The base of each lesion was sent to histological evaluation separately with a numerical code to compare histological results with those of cystoscopy and mp-MRI. Once the muscle-invasiveness was histologically proved, patients underwent to radical cystectomy. Since they were eligible, they also received a platinum-based neoadjuvant chemotherapy [21]. When indicated, a second TURB was performed according to the EAU guidelines [4]. Specimens were examined to assess the type, grade, and stage of the tumor. The World Health Organization classification was used to classify and grade malignant tumors, while the American Joint Committee on Cancer/Union for International Cancer Control TNM system was used to stage each lesion [4].

### Statistical analysis

The Shapiro-Wilk test was used to test the distribution of quantitative variables showing a non-normal distribution. Sample size estimation was performed considering the association between VI-RADS score and MIBC status. The reference was the proportion of NMIBC and MIBC stratified according to VI-RADS score in previous studies [14, 19]. A power of 90% and a value of 5% were considered for the *χ*^2^ test. Sample size was estimated by using the R package “pwr” (version 1.2.2; function: pwr.chisq.test). The estimated effect size was 0.7, and the number of patients needed to obtain the desired power was 31 subjects. A dichotomization of VI-RADS scores was performed. In detail, concerning the diagnostic accuracy for the detection of MIBC, VI-RADS scores of 1–3 were considered negative for muscle-invasiveness, while VI-RADS scores of 4 and 5 were considered positive.

Descriptive statistics, including sensitivity and specificity, were calculated for each reader. Receiver operating characteristic (ROC) analysis was performed, and areas under the curve (AUCs) were calculated for each reader and image set. A comparison of ROC curves was performed to test the difference between the areas under the ROC curves among the four readers. ROC curve comparison was performed with MedCalc software, version 16.8.4 (MedCalc Software). All other statistical tests were performed by using IBM SPSS Statistics software, version 20 (IBM). A *p* value ≤ 0.05 was considered statistically significant.

## Results

Most of the 38 patients included in the study were male (27, 71.1%), and the median age was 72.5 (IQR: 66.5–81.0) years (Table 2). Out of the 38 patients, 7 (18%) had a MIBC (T2–T3), and 31 (82%) had a NMIBC (Ta–T1). A total of 68 BCs were found. More in detail, 33 (48.5%) were Ta, 28 (41.2%) were T1, 6 (8.8%) were T2W, and 1 (1.5%) was T3. All readers correctly classified the 7 MIBC patients with both MRI protocols.

**Table 2:** Descriptive baseline characteristics of included patients (*n* = 38). Continuous variables are summarized as median and interquartile ranges (IQR). Categorical variables are summarized as frequencies and percentages (%)

Features	Value
Age	72.5 (66.5, 81.0)
Gender, male	27 (71.4%)
Body mass index, kg/m^2^	26.6 (24.0, 29.1)
Smoking history	14 (36.8%)
Previous bladder cancer	19 (50.0%)
Charlson Comorbidity Index	
0	21 (55.3%)
1	13 (34.2%)
2	3 (7.9%)
3	1 (2.6%)
Urine cytology	
Non-diagnostic	13 (34.2%)
Negative	16 (42.1%)
Positive	9 (23.7%)
Previous endovesical treatment	
Bacillus Calmette-Guerin	4 (10.5%)
Epirubicin	1 (2.6%)
Mitomycin C	3 (7.9%)
None	30 (78.9%)

The diagnostic performance of the readers referring to descriptive statistics and ROC analysis is shown in Table 3. More in detail, no significant differences were shown in diagnostic performance for all readers between the two protocols (*p* > 0.05). In fact, the AUC for the bp- and mp-MRI protocol was respectively 0.91–0.92 (reader 1), 0.90 (reader 2), 0.95–0.90 (reader 3), and 0.90–0.87 (reader 4). No significant differences in diagnostic performance were found among the four readers in the pairwise comparisons (*p* > 0.05).

**Table 3:** Diagnostic performance of the four readers regarding the MIBC detection for image set 1 and image set 2

	MIBC detection							Pairwise ROC curve comparison		
	Sensitivity (95% CI)		Specificity (95% CI)		AUC set 1 (95% CI)	AUC set 2 (95% CI)	*p* value	Pairwise readers	*p* value set 1	*p* value set 2
	Set 1	Set 2	Set 1	Set 2						
Reader 1	100.00 (59.04–100.00)	100.00 (59.04–100.00)	82.76 (64.23–94.16	83.33 (65.28–94.36)	0.91 (0.77–0.98)	0.92 (0.78–0.98)	0.99	Reader 1 vs reader 2 Reader 1 vs reader 3 Reader 1 vs reader 4 Reader 2 vs reader 3 Reader 2 vs reader 4 Reader 3 vs reader 4	0.66 0.42 0.99 0.17 0.57 0.42	0.57 0.66 0.32 0.99 0.57 0.71
Reader 2	100.00 (59.04–100.00)	100.00 (59.04–100.00)	79.31 (60.28–92.01)	79.31 (60.28–92.01)	0.90 (0.75–0.97)	0.90 (0.75–0.97)	0.99			
Reader 3	100.00 (59.04–100.00)	100.00 (59.04–100.00)	89.66 (72.65–97.81)	79.31 (60.28–92.01)	0.95 (0.82–0.99)	0.90 (0.75–0.97)	0.07			
Reader 4	100.00 (59.04–100.00)	100.00 (59.04–100.00)	80.00 (61.43–92.29)	74.19 (55.39–88.14)	0.90 (0.76–0.97)	0.87 (0.77–0.96)	0.15			

**Fig. 2:**
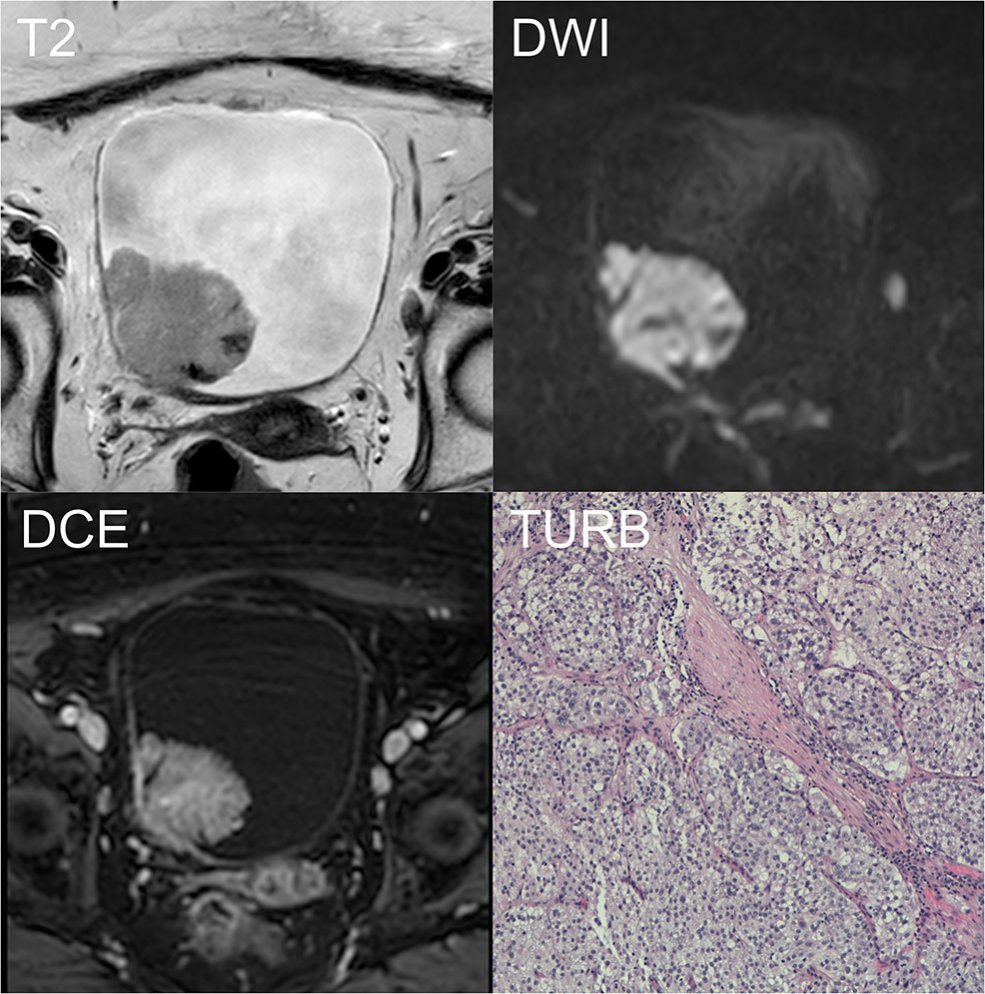
Example of correctly classified muscle-invasive BC. A 90-year-old woman with hematuria and a bladder mass reported after flexible cystoscopy underwent mp-MRI before primary TURB. T2W imaging (T2) showing an exophytic lesion on the right lateral wall, > 1 cm in the major axis with focal interruption of the SI of the muscularis propria. The *b*1000-DWI (DWI) confirming the high signal intensity of the tumor extending to the muscular layer. Based on bp-MRI, all readers assigned a VI-RADS category of 4. DCE imaging (DCE) showing early enhancement of the lesion and inner layer with an early enhancement of the muscularis propria, indicating tumor infiltration. DCE did not affect the VI-RADS category. T stage after TURB was HG-T2 (TURB). DCE, dynamic contrast-enhanced; DWI, diffusion-weighted imaging; HG, high grade; mp-MRI, multiparametric magnetic resonance imaging; bp-MRI, biparametric magnetic resonance imaging; SI, signal intensity; T2W, T2-weighted; TURB, trans-urethral resection of the bladder; VI-RADS, Vesical Imaging-Reporting and Data System

**Fig. 3:**
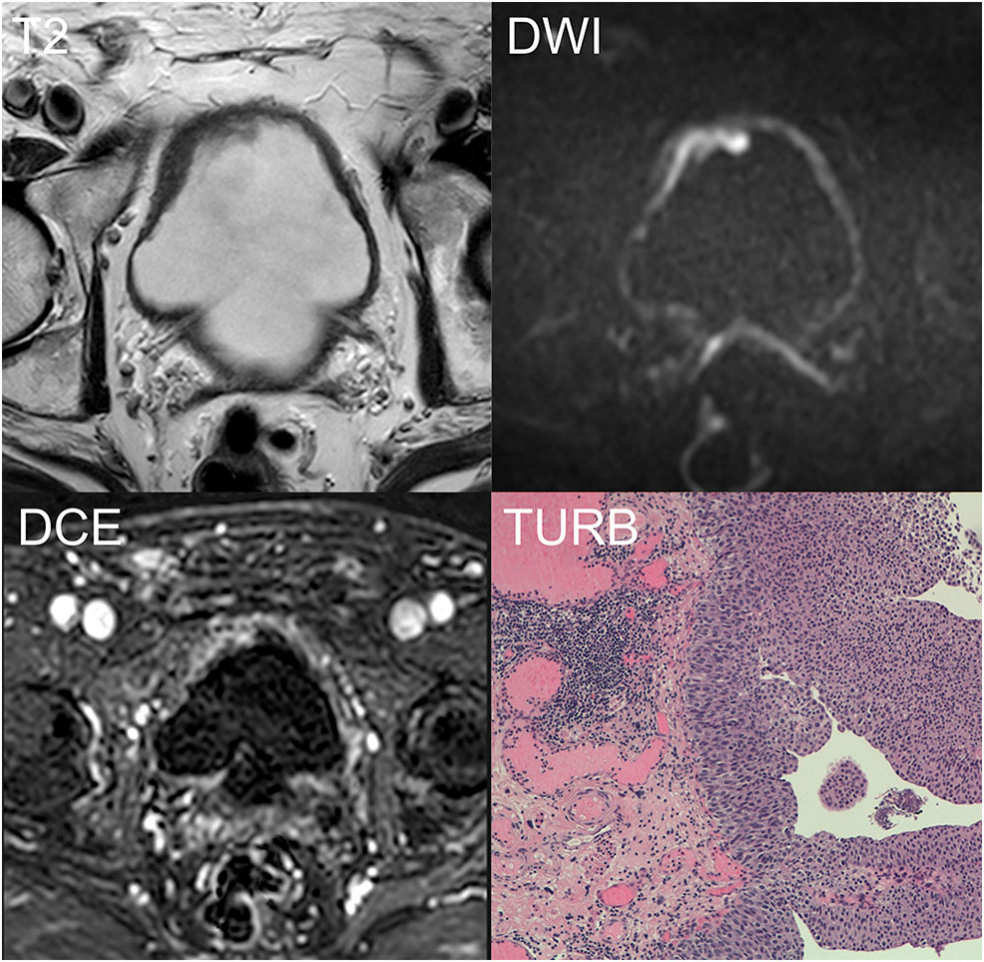
Example of incorrectly classified not muscle-invasive BC. An 89-year-old man with hematuria and bladder mass reported after flexible cystoscopy underwent mp-MRI before primary TURB. T2W imaging (T2) shows a slightly exophytic lesion on the right antero-lateral wall, which is 1 cm thick with equivocal interruption of the SI of the muscularis propria. The *b*1000-DWI (DWI) clearly demonstrated the continuous intermediate signal intensity of the muscular layer. Based on bp-MRI, three of the four readers assigned a VI-RADS category of 3. DCE imaging (DCE) showing early enhancement of the lesion with an inhomogeneous enhancement of the underlying inner layer due to inflammatory changes. This finding was misinterpreted by two readers as the muscularis propria infiltration, thus modifying the VI-RADS category from 3 to 4. T stage after TURB was LG-T1 (TURB). After 4 weeks, re-TURB was performed confirming the absence of residual tumor. DCE, dynamic contrast-enhanced; DWI, diffusion-weighted imaging; LG, low grade; mp-MRI, multiparametric magnetic resonance imaging; bp-MRI, biparametric magnetic resonance imaging; SI, signal intensity; T2W, T2-weighted; TURB, trans-urethral resection of the bladder; re-TURB, repeated trans-urethral resection of the bladder; VI-RADS, Vesical Imaging-Reporting and Data System

Table 4 shows a per-lesion diagnostic performance analysis for each reader to classify MIBC according to the TNM stage classification correctly. Comparing the false positive cases between bp-MRI and mp-MRI, they reduced for reader 1 (from 7 to 6), were stable for reader 2 (7), and increased for the two less experienced readers when using the mp-MRI (from 4 to 7 for reader 3, from 8 to 10 for reader 4). The number of false negative cases remained stable for readers 2 and 3 (6 cases for both readers) and reduced when using the mp-MRI for readers 1 and 4 (from 7 to 6 and from 2 to 1, respectively). Case examples of correctly and incorrectly classified MIBC are shown in Figs. 2 and 3, respectively.

**Table 4:** Per-lesion diagnostic performance of the four readers to correctly classify MIBC according to TNM stage classification

		Biparametric bladder MR			Triparametric bladder MR		
		Correctly classified	Incorrectly classified		Correctly classified	Incorrectly classified	
			False negatives	False positives		False negatives	False positives
Ta (*n* = 33)	Reader 1	25	5	3	26	4	3
	Reader 2	26	3	4	26	3	4
	Reader 3	26	3	4	25	3	5
	Reader 4	27	2	4	26	1	6
T1 (*n* = 28)	Reader 1	24	0	4	25	0	3
	Reader 2	22	3	3	22	3	3
	Reader 3	25	3	0	23	3	2
	Reader 4	24	0	4	24	0	4
T2W (*n* = 6)	Reader 1	6	0	0	6	0	0
	Reader 2	6	0	0	6	0	0
	Reader 3	6	0	0	6	0	0
	Reader 4	6	0	0	6	0	0
T3 (*n* = 1)	Reader 1	1	0	0	1	0	0
	Reader 2	1	0	0	1	0	0
	Reader 3	1	0	0	1	0	0
	Reader 4	1	0	0	1	0	0

## Discussion

Our results demonstrate that a contrast-free MR protocol of the bladder has the same diagnostic accuracy of the standard mp-MRI for assessing BC’s muscle-invasiveness, regardless of the reader’s experience.

These results firstly confirm the relevance of DWI in the risk evaluation of muscle-invasiveness in bladder cancer [8]. In detail, the diagnostic accuracy of the bp-MRI protocol is in line with what is reported by several other studies [22–26]. Secondly, our results support the promising results of Barchetti et al, on the VI-RADS repeatability [11]. In fact, in our study, the diagnostic performance of all readers was substantially comparable, demonstrating that VI-RADS is a useful tool even in less experienced readers.

An interesting finding was the worse diagnostic performance of the two less experienced readers (readers 3 and 4) when including DCE imaging. On the other hand, the diagnostic performance of the two most experienced readers was not significantly affected between the two protocols. More in detail, focusing on the per-lesion muscle-invasiveness assessment (Table 4), we observed that, while the overall number of false negative cases remained stable among the four readers, the overall number of false positive cases increased in most readers when adding DCE imaging. The overall increase was 15.4% (from 26 to 30) in line with what is reported in other studies [22, 27, 28]. However, considering only readers 3 and 4, it reached 41.7% (from 12 to 17), implying that DCE imaging should be interpreted carefully by not-expert readers. We believe there are two possible reasons. First, we postulated that DWI imaging could require a relatively more straightforward learning curve compared to DCE imaging. For instance, the general principle of DWI is relatively straightforward: the tumor appears as a hyperintense signal interrupting or not the submucosal muscle, which has a typical low-intermediate signal intensity. Moreover, as demonstrated in pelvic tumors, our DWI protocol includes high *b*-values, which could have improved the diagnostic confidence of the less experienced readers by providing a better background suppression [29, 30]. Secondly, it is well known that, in approximately 60% of DCE studies, the submucosa and the tumor show similar signal intensity, thus making more challenging the detection of the submucosal linear enhancement [31]. Moreover, perivesical enhancement due to reactive or acute inflammatory changes, for instance, in case of concomitant cystitis, may be a confounding factor potentially leading to an overestimation of the tumor stage [28, 32].

Despite these results, the mp-MRI protocol reduced the overall number of false negative cases among the four readers (from 21 to 19). In detail, only adding DCE, two readers recognized a missed 3-mm Ta BC. In this regard, further studies are necessary to investigate the added value of DCE in < 5-mm lesions.

A potential downside of bp-MRI of the bladder is that DWI has few potential pitfalls that may affect the diagnostic accuracy [33]. For example, the presence of edema can be responsible for high signal intensity on DWI due to the intrinsic long T2W decay time of water (also known as “shine-through” effect) [34]. To overcome this problem, we recommend using multiple *b*-values including middle high *b*-values (*b*800–*b*1000) and very high *b*-values (*b*1500–*b*2000), where the shine-through effect is more likely to be absent, and to include ADC map on visual analysis.

Although it is a relatively novel topic in bladder cancer, the value of a contrast-free protocol was already investigated in the pelvis. For example, Di Campli et al reported no significant differences between the standard multiparametric and biparametric (T2W and DWI) MRI for clinically significant prostate cancer detection [16]. Concerning rectal cancer, the 2016 consensus meeting of the European Society of Gastrointestinal and Abdominal Imaging recommended the crucial role of DWI in the staging and the re-staging after chemoradiotherapy and before surgery [15, 35].

To the best of our knowledge, this is the first study comparing the diagnostic accuracy of bladder bp-MRI and mp-MRI for the muscle-invasiveness assessment using VI-RADS in a clinical setting. Most of the recent studies focused on standard mp-MRI and the feasibility of VI-RADS [11–13]. In addition, our multi-reader approach may expand the applicability of our results to clinical settings with less experienced readers. In light of the emerging role of VI-RADS and its potential future extension to the surveillance and treatment response assessment, the results of our study may have a beneficial impact on costs, scan time, and patient safety [36, 37].

Overall, from a clinical point of view, it should be pointed out that currently to think to a patient management relying only on MRI findings is not realistic. In fact, although we had no “false negative” muscle-invasive bladder cancers in our study, this event may produce a rapid progression and spread of the tumor with devastating consequences for the patient. Similarly, to perform a radical cystectomy in a “false positive” muscle-invasive bladder cancer would be a failure as well, especially considering the high comorbidity rate of this surgical treatment. For these reasons, as recently discussed by Marchioni et al, a probable scenario would be to consider MRI in a more complex and articulate approach to the patient, in order to reduce useless cystoscopy or re-TURB [14].

Our study has some limitations. First of all, the type of VI-RADS scores dichotomization could have represented a bias. In fact, the choice of the appropriate VI-RADS category to define whether the tumor is muscle-invasive is controversial [14, 38]. Marchioni et al recently demonstrated that a threshold of 4 significantly improved MIBC detection, reaching an accuracy of 90%. Moreover, the type of dichotomization and the relatively small study cohort could have affected the sensitivity, which is higher than other studies [14, 38, 39]. Secondly, patient selection in the study was widely influenced by physician experience and patient characteristics. In this way, patients with worrisome tumor features (i.e., large and solid tumors) may have not been included to avoid delaying cystectomy. Third, ours is a single-center study. However, all the studies recently published involved a single center. Therefore, further studies, possibly including multiple institutions, shall represent the next step for future standardization [11, 14, 19, 38, 39]. Nonetheless, we used a state-of-the-art 3-T MR scanner for all patients, thus setting up a good standard for future investigation. In fact, 3-T MRI has been proven to provide better specificity and sensitivity for the bladder cancer T staging [40]. Fourth, we know that a certain degree of unbalanced data (patients and lesions, MIBC vs. NMIBC) could have affected our results. However, considering the purpose of this study, our primary objective was to test if any difference in MIBC detection at biparametric MRI vs. multiparametric MRI exists. In this way, all lesions were examined with both protocols by different readers. Moreover, both a per-patient and a per-lesion analyses were performed for each reader.

## Conclusion

A contrast-free MR imaging protocol consisting of T2W and DWI has comparable diagnostic accuracy to that of a standard mp-MR imaging protocol for the detection of MIBC. Moreover, considering both MR protocols, the experience of the reader does not significantly affect the diagnostic performance when using VI-RADS classification. Further validation studies in a larger population are warranted to define better the clinical role of bp-MRI, and whether it could be considered an accurate alternative to the standard protocol definitively.

## Abbreviations

ADC: Apparent diffusion coefficient
BC: Bladder cancer
bp-MRI: Biparametric magnetic resonance imaging
DCE: Dynamic contrast-enhanced
DWI: Diffusion-weighed imaging
MIBC: Muscle-invasive bladder cancer
mp-MRI: Multiparametric magnetic resonance imaging
NMIBC: Non-muscle-invasive bladder cancer
T2W: T2W-weighted images
TURB: Trans-urethral resection of bladder
VI-RADS: Vesical Imaging-Reporting and Data System
